# Supplementary material for: Facile Synthesis of Quinolinecarboxylic Acid–Linked Covalent Organic Framework via One–Pot Reaction for Highly Efficient Removal of Water–Soluble Pollutants
Source: Molecules. 2023 Apr 27;28(9):3752. doi: 10.3390/molecules28093752 (PMC10179942; doi:10.3390/molecules28093752)
Supplement: Supplementary file 1 [file molecules-28-03752-s001.zip › molecules-2376368-supplementary.pdf]

Supplementary Materials

Methods

Characterization. Scanning electron microscope (SEM) images were recorded on a SWPRATM55 scanning electron microscope (Carl Zeiss, AG, Aalen, Germany). The FT-IR data were obtained ranging from 500 to 3700 cm^{-1} on a Nicolet 710 spectrometer (California, USA). The N_2 adsorption-desorption experiments were performed on a Kubo-X1000 analyzer (Bjbuilder, Beijing, China). The Brunauer−Emmett−Teller (BET) method was applied to calculate the surface area and pore volume. The pore size distribution was calculated with the Barrett−Joyner−Halenda model. Thermogravimetric (TG) analysis was performed from room temperature to 850 °C on the STA 449F3-QMS403C system (Netzsch, Germany). The X-ray photoelectron spectroscopy (XPS) was conducted using an ESCALAB 250XI imaging electron spectrometer (Thermo, Waltham, MA, USA). A Vario EL III analyzer (Elementar, Germany) was used to perform the elemental analysis.

The liquid-state NMR was recorded on an Avance III HD 400 MHz spectrometer (Bruker Biospin AG, Switzerland) using tetramethylsilane (TMS) as an internal standard. Chemical shifts were reported in ppm. ^{1}H NMR spectra were referenced to DMSO-d_6 (2.50 ppm), and ^{13}C-NMR spectra were referenced to DMSO-d_6 (39.5 ppm). All ^{13}C NMR spectra were measured with complete proton decoupling. The solid-state ^{13}C NMR spectra were measured on a JNM-ECZ600R NMR spectrometer (JEOL, Japan). Contaminants were analyzed using the Waters ACQUITY UHPLC system coupled to a Waters Xevo TQ-XS triple quadrupole mass spectrometer (Milford, USA).

The conditions of UHPLC-MS/MS. The analysis of four contaminants was performed using a Waters Acquity UPLC coupled with Waters Xevo TQ-XS (Milford, USA). The separations were obtained using a Waters Acquity UPLC BEH C18 column (1.7 µm, 2.1 × 50 mm) with the mobile phase as listed in Table S4 and Table S3 pumped at a flow rate of 0.3 mL/min. The temperature of auto sampler and column were 10 °C and 40 °C, respectively. The injection volume was 5 µL.

The mass spectrometry experiment was working with ESI mode under the multiple reaction monitoring (MRM) conditions. The MRM details are listed in Table S5. The ESI source parameters were: capillary voltage, 0.5 kV; source temperature, 150 ^{o}C; desolvation temperature, 400 ^{o}C; desolvation gas, 800 L h^{-1}; desolvation gas, 99.99% purity of N_2; nebulization flow, 7.0 Bar.

The linear ranges and limits of quantification (LOQs) for the determination of four contaminants by UHPLC-MS/MS are listed in Table S6.

The synthesis of quinoline-4-carboxylic acid. A mixture of pyruvic acid (106 mg, 1.2 mmol), aniline (102 mg, 1.1 mmol), and benzaldehyde (106 mg, 1.0 mmol) in 1,4-dioxane/*n*-butanol solution (5 mL, *v/v* = 1:4) was well stirred with NH_2SO_3H (3 mg, 3 mol%). This mixture was magnetically stirred under 100 ^{o}C for 4h. Then, the reaction mixture was cooled to room temperature and concentrated to dryness in *vacuo*. The residue was recrystallized using ethanol to obtain quinoline-4-carboxylic acid as white solid (189 mg, 76%). ^{1}H NMR (400 MHz, d6-DMSO): δ 14.03 (s, 1H), 8.69 (d, 1H, J = 8.0 Hz), 8.49 (s, 1H), 8.32 (d, 1H, J = 8.0 Hz), 8.19 (d, 2H, J = 8.0 Hz), 7.88 (t, 1H, J = 8.0 Hz), 7.73 (t, 1H, J = 7.2 Hz), 7.53-7.63 (m, 3H).

Isothermal adsorption. The working solutions (10–200 μg/mL) for 2,4-D, MB, RhB, and gentamicin were prepared. QCA-COF (10 mg) was added to each solution (20.0 mL). The mixture was shaken for 45 min at 25 °C. After adsorbing, the free concentration of 2,4-D, MB, RhB, and gentamicin was measured by UHPLC-MS/MS. The quantity (Q_e, mg/g) of adsorbed to QCA-COF was calculated by the following equation (1):

$$Q_e = (C_o - C_e)\, v \,/\, m \qquad (1)$$

where C_o (μg/mL) is the initial concentration, C_e (μg/mL) is the equilibrium concentration, v (mL) is the volume of water sample, and m (mg) is the mass of QCA-COF.

The adsorption data are described by Freundlich equation (2) and Langmuir equation (3) as following:

$$lnQ_e = lnC_e \,/\, n + lnK_F \qquad (2)$$

$$C_e \,/\, Q_e = 1 \,/\, Q_m K_L + C_e \,/\, Q_m \qquad (3)$$

For Freundlich equation, n is the indicator of adsorption intensity, while K_F is the indicator of adsorption capacity. According to the Freundlich theory, it is favorable adsorption when $n > 1$. For Langmuir equation, Q_m is the theoretical maximum adsorption capacity.

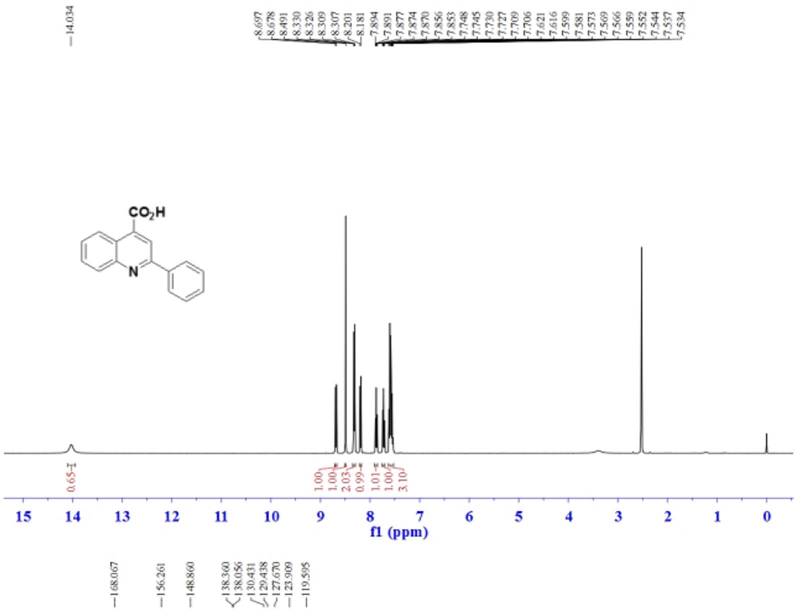

Figure S1. ^{1}H NMR spectrum of model compound.

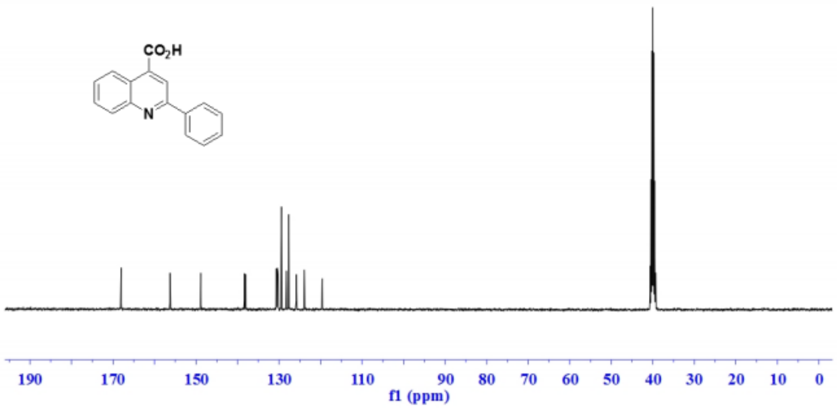

Figure S2. ^{13}C NMR spectrum of model compound.

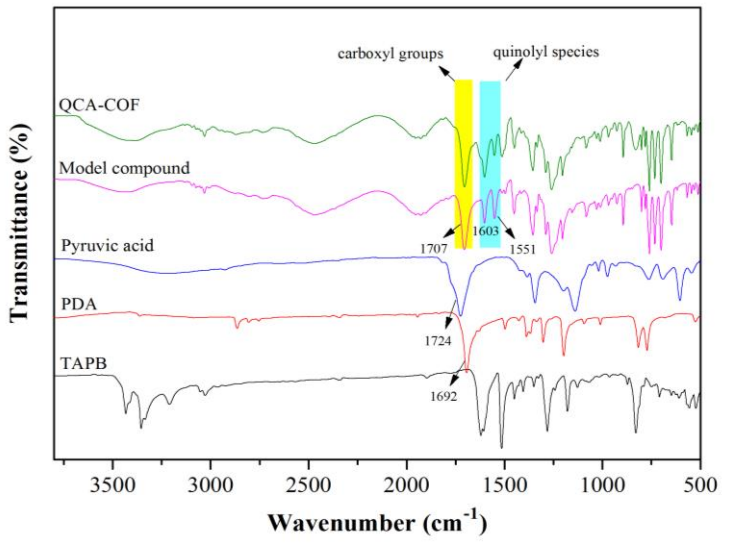

Figure S3. FT-IR spectra of TAPB, PDA, pyruvic acid, model compound, and QCA-COF.

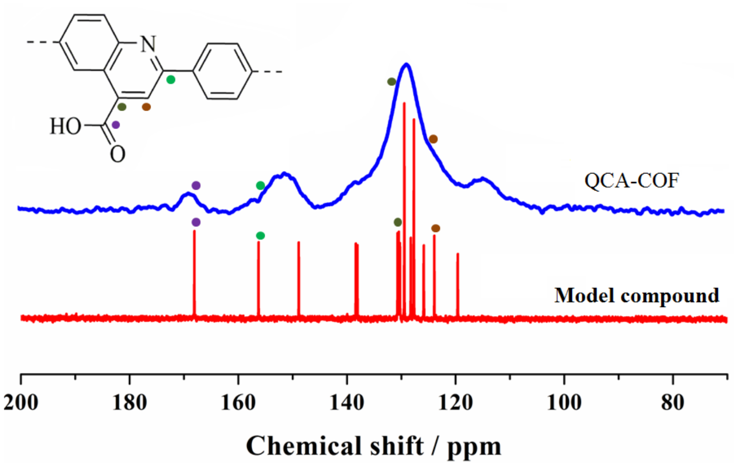

Figure S4. Liquid-state ^{13}C NMR spectrum of model compound and solid-state ^{13}C NMR spectra of QCA-COF.

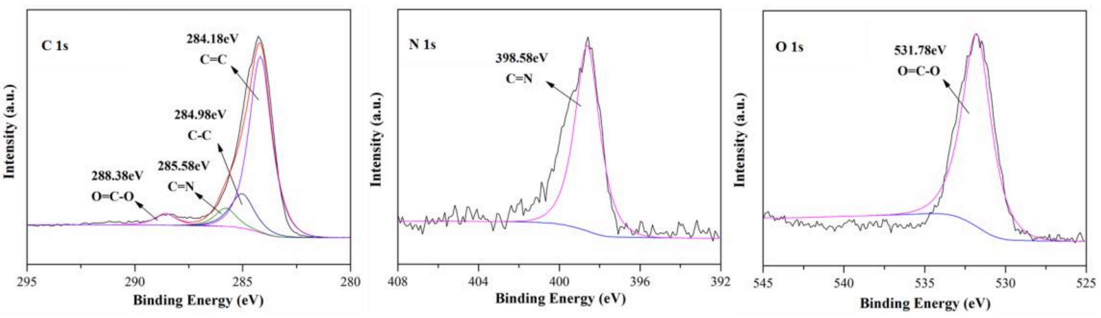

Figure S5. High-resolution XPS spectra of C, N, and O for QCA-COF.

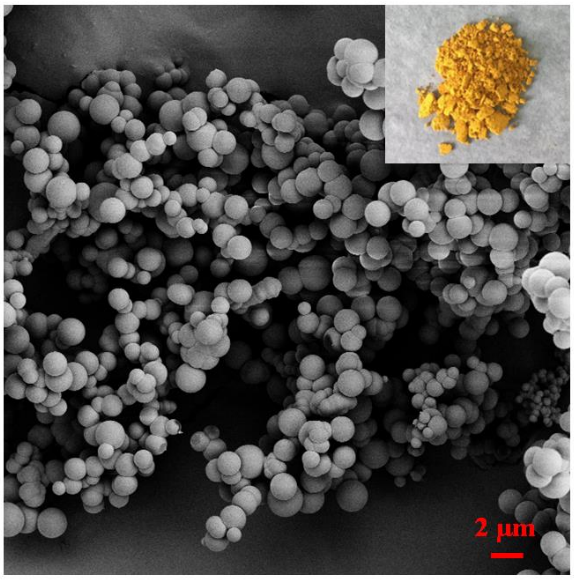

Figure S6. SEM image of QCA-COF, Inset showing its appearance.

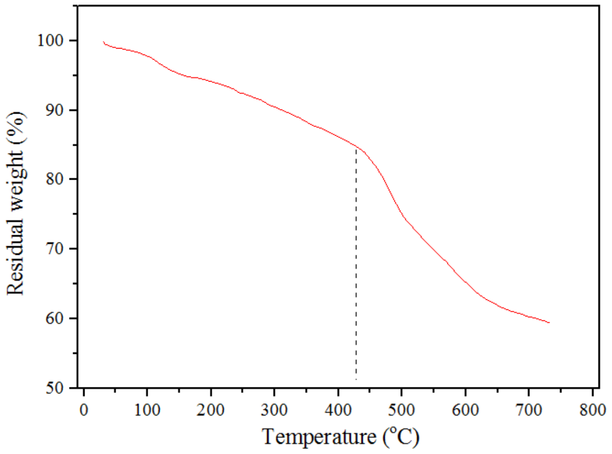

Figure S7. TGA data indicate that QCA-COF is thermally stable up to 420 °C.

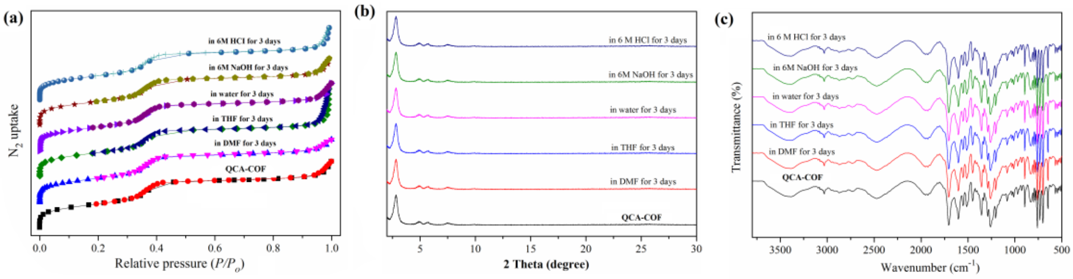

Figure S8. N_2 adsorption-desorption isotherms (a), PXRD patterns (b) and FT-IR spectra (c) for QCA-COF measured after 72 h treating with DMF, THF, water, 6M NaOH, and 6M HCl.

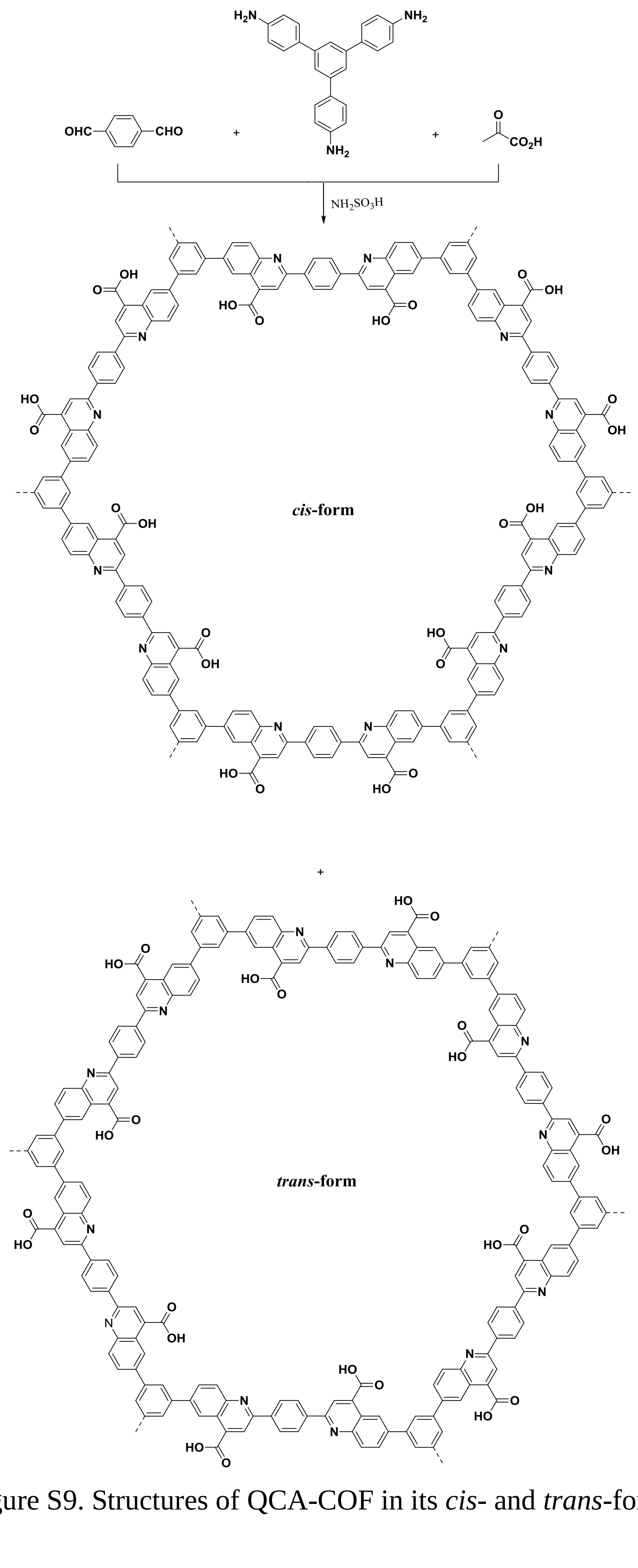

Figure S9. Structures of QCA-COF in its *cis*- and *trans*-form.

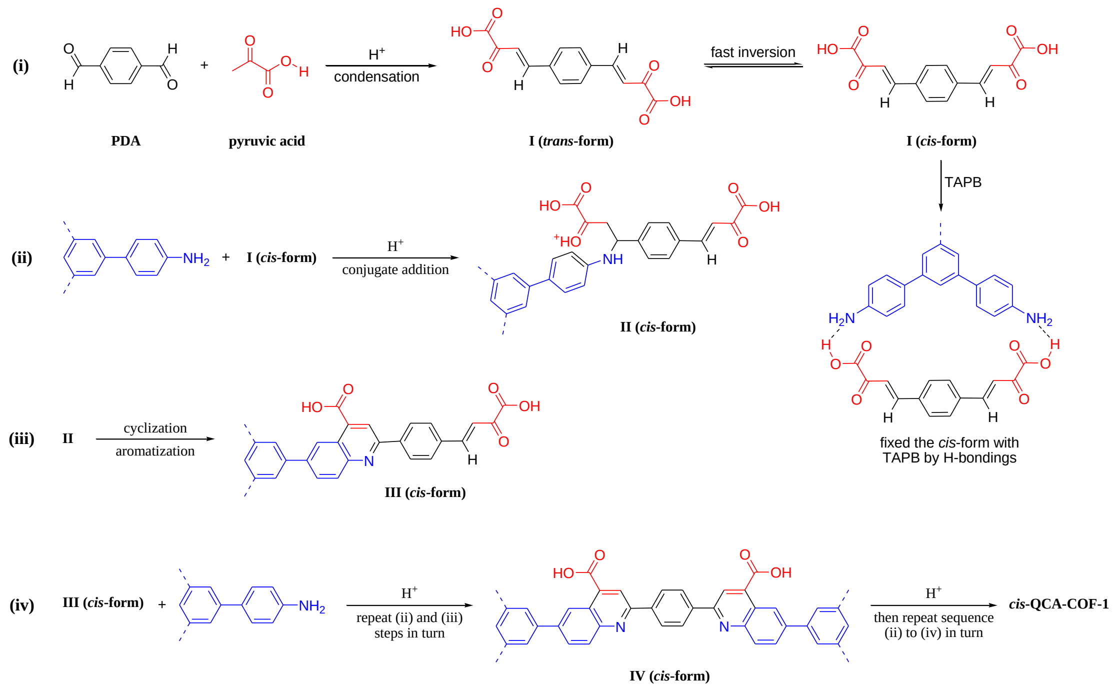

Figure S10. A plausible mechanism proposed for QCA-COF yielded in its *cis*-form.

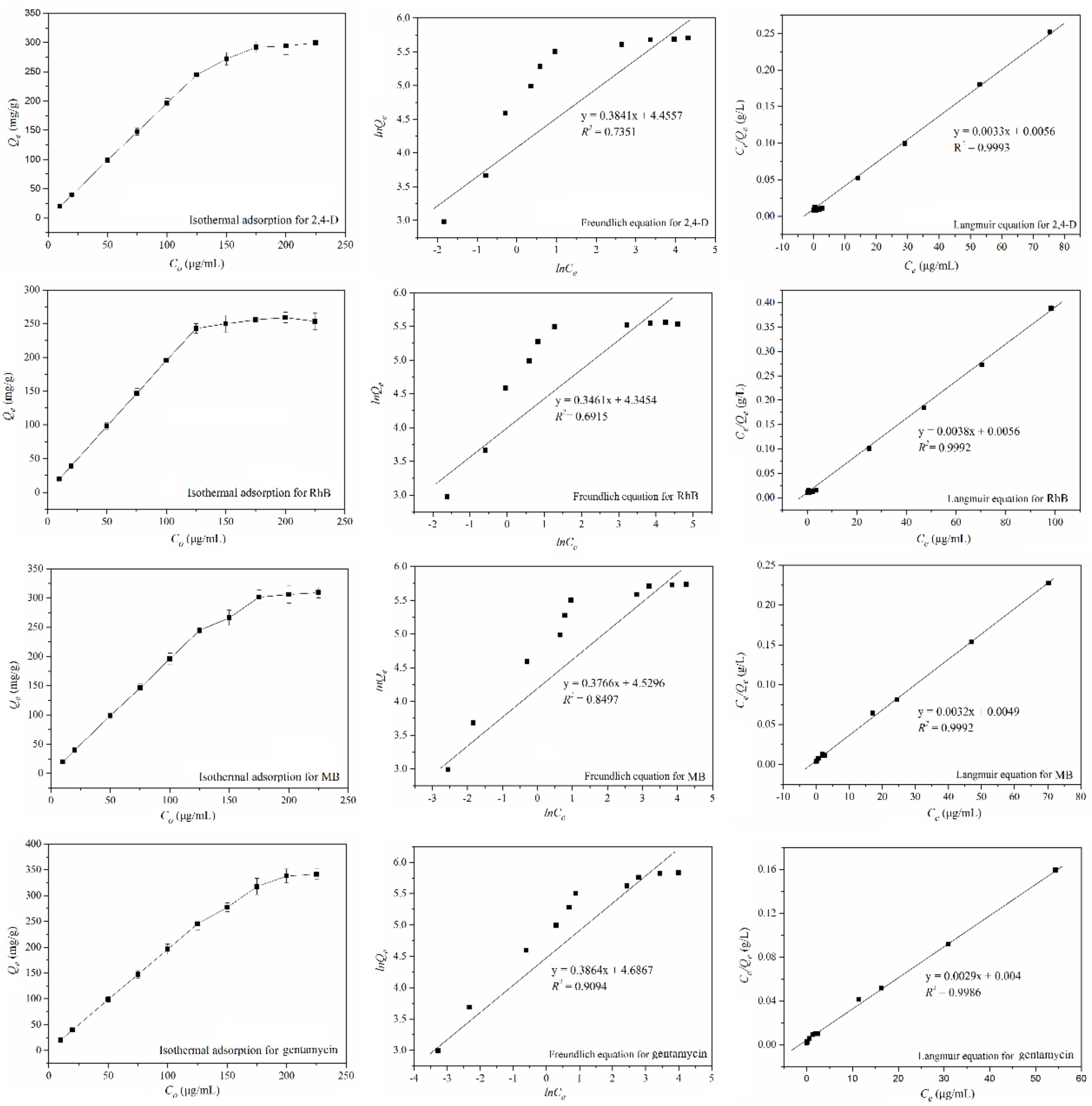

Figure S11. The isothermal adsorption, Langmuir model, and Freundlich model of four contaminants binding by QCA-COF.

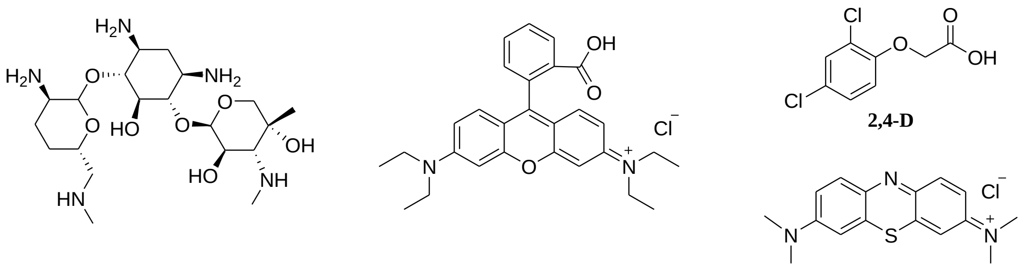

Gentamycin **RhB** **2,4-D** **MB**

Figure S12. Chemical structures of four water-soluble organic contaminants.

Table S1. The optimized conditions by changing solvents and temperatures.

Solvent	The ratio of solvent	Temp. (°C)	Time (h)	Crystallinity
o-DCB	-	100	8	Amorphous
n-Butanol	-	100	8	Poor
o-DCB/*n*-Butanol	1:1	110	8	Amorphous
Dioxane	-	100	8	Amorphous
Dioxane/*n*-Butanol	1:1	100	8	Amorphous
Mesitylene	-	100	8	Amorphous
Mesitylene/*n*-Butanol	1:1	100	8	Amorphous
Dioxane/*n*-Butanol	1:2	100	8	Poor
Dioxane/*n*-Butanol	1:3	100	8	Poor
Dioxane/*n*-Butanol	1:4	100	8	Poor
Dioxane/*n*-Butanol	1:4	110	8	Crystallinity
Dioxane/*n*-Butanol	1:4	120	8	Poor
Dioxane/*n*-Butanol	4:1	110	8	Amorphous
Dioxane/*n*-Butanol	3:1	110	8	Amorphous
Dioxane/*n*-Butanol	2:1	110	8	Amorphous

Table S2. Unit cell parameters and fractional atomic coordinates for QCA-COF in *cis*-form based AA topology after unit cell correction and Pawley refinement.

Element	Atom Number	Hexagonal, P6. Fractional coordinates of atoms (a = 35.78 Å, b = 36.58 Å, c = 3.60 Å; α = 89.50°, β = 90.61°, γ = 119.65°)		
H	1	3.88474	-0.25324	0.81658
H	2	3.99024	-0.12823	0.9649
H	3	3.8706	-0.14772	0.50619
H	4	4.27838	0.43457	0.08625
H	5	4.29276	0.55183	0.43783
H	6	4.17143	0.45638	2.24E-04
H	7	4.0169	-0.07568	0.55259
H	8	4.04812	-0.0011	0.40951
H	9	3.89404	-0.08351	0.74264
H	10	3.91771	0.0511	0.33859
H	11	4.06807	0.11522	0.39263
H	12	4.1062	0.19105	0.30336
H	13	3.98766	0.18631	-0.02823
H	14	3.94942	0.1106	0.04917
H	15	4.02122	0.24814	0.33696
H	16	4.19347	0.30383	-0.23903
H	17	4.23848	0.37975	-0.24663
H	18	4.13488	0.3928	0.20521
H	19	3.86182	0.01161	0.73905
H	20	4.05905	0.37964	0.67094
H	21	3.92502	-0.27005	1.16847
H	22	3.97185	-0.30086	1.15452
H	23	4.02641	-0.15492	0.71249
H	24	4.14138	-0.18522	0.54566
H	25	4.06117	-0.32795	0.89834
H	26	4.09874	-0.36764	0.84947
H	27	4.21492	-0.25341	0.55185
H	28	4.17927	-0.21387	0.62963
H	29	4.24732	-0.28792	0.70356
H	30	4.11434	-0.4644	0.56818
H	31	4.14475	-0.51007	0.46744
H	32	4.27019	-0.40071	0.32429
H	33	4.34486	-0.3306	0.78897
H	34	4.1613	-0.08336	0.44434
H	35	4.81108	0.80123	0.80859
H	36	4.73461	0.75626	0.68796
H	37	4.83509	0.70249	0.45609

H	38	4.6921	0.58908	0.16482
H	39	4.62479	0.66481	0.12064
H	40	4.54681	0.6255	0.09903
H	41	4.54955	0.51511	0.50951
H	42	4.62733	0.55446	0.51723
H	43	4.48287	0.48083	0.35202
H	44	4.43159	0.60819	0.13033
H	45	4.35399	0.57818	0.10415
H	46	4.33754	0.45765	0.4284
H	47	4.73963	0.57159	0.46788
H	48	4.34811	0.38097	0.13102
C	1	3.87124	-0.20351	0.68447
C	2	3.89794	-0.2197	0.79785
C	3	3.94183	-0.19288	0.87784
C	4	3.95783	-0.1492	0.86947
C	5	3.93282	-0.13223	0.72978
C	6	3.8897	-0.1599	0.63407
C	7	4.22037	0.43876	0.06041
C	8	4.26471	0.45473	0.12225
C	9	4.29168	0.49656	0.23384
C	10	4.27305	0.52157	0.30946
C	11	4.2295	0.50762	0.22855
C	12	4.20408	0.46665	0.08823
C	13	3.95245	-0.08617	0.65391
C	14	3.9963	-0.06186	0.56278
C	15	4.01419	-0.01933	0.47301
C	16	3.98825	-8.05E-04	0.45081
C	17	3.94386	-0.0249	0.52109
C	18	3.92696	-0.06663	0.64637
C	19	3.98236	0.06025	0.32269
C	20	3.93756	0.03672	0.37343
C	21	3.91785	-0.00624	0.46349
C	22	3.87015	-0.03166	0.46095
C	23	4.00515	0.10631	0.24109
C	24	4.04995	0.13008	0.29808
C	25	4.07194	0.17362	0.24664
C	26	4.04971	0.19465	0.13972
C	27	4.00536	0.17103	0.06745
C	28	3.98335	0.12736	0.11495
C	29	4.07294	0.24112	0.11508
C	30	4.13841	0.3042	-0.00726
C	31	4.12103	0.32915	0.1184

C	32	4.0776	0.30853	0.24143
C	33	4.05386	0.2644	0.23287
C	34	4.05556	0.33097	0.38342
C	35	4.18051	0.32307	-0.13786
C	36	4.20634	0.36666	-0.13756
C	37	4.19081	0.39255	-6.20E-04
C	38	4.14752	0.37327	0.11367
C	39	3.97177	-0.21017	0.92697
C	40	3.95714	-0.25131	1.05953
C	41	3.98388	-0.269	1.05388
C	42	4.02578	-0.24603	0.91842
C	43	4.04192	-0.20421	0.79319
C	44	4.01471	-0.18664	0.80581
C	45	4.09195	-0.244	0.78048
C	46	4.10942	-0.20208	0.65877
C	47	4.08488	-0.18177	0.6614
C	48	4.11636	-0.2674	0.76231
C	49	4.09494	-0.31112	0.82752
C	50	4.11651	-0.33407	0.79772
C	51	4.16018	-0.31412	0.70002
C	52	4.18173	-0.27037	0.63812
C	53	4.16044	-0.24735	0.67449
C	54	4.18201	-0.3395	0.64912
C	55	4.17523	-0.40695	0.54954
C	56	4.2201	-0.38903	0.50991
C	57	4.24651	-0.34547	0.57607
C	58	4.22707	-0.32094	0.64202
C	59	4.14865	-0.45047	0.52645
C	60	4.16619	-0.47644	0.46063
C	61	4.2105	-0.45913	0.39041
C	62	4.23683	-0.41504	0.40451
C	63	4.2939	-0.32543	0.61374
C	64	4.10586	-0.13771	0.52
C	65	4.82942	0.75573	0.62921
C	66	4.80028	0.77023	0.69546
C	67	4.75643	0.7445	0.63056
C	68	4.74055	0.70351	0.50332
C	69	4.76881	0.68784	0.43175
C	70	4.81321	0.71427	0.49933
C	71	4.67953	0.63787	0.34674
C	72	4.70619	0.62095	0.26783
C	73	4.75108	0.64573	0.30575

C	74	4.63215	0.61258	0.31481
C	75	4.60852	0.63197	0.20172
C	76	4.5637	0.60929	0.1903
C	77	4.54107	0.56662	0.2972
C	78	4.56502	0.54727	0.41177
C	79	4.60999	0.56994	0.41776
C	80	4.49303	0.54405	0.29874
C	81	4.77803	0.62675	0.20565
C	82	4.42932	0.54934	0.2497
C	83	4.40284	0.50575	0.31032
C	84	4.42268	0.48049	0.3415
C	85	4.4679	0.5002	0.33925
C	86	4.39809	0.43375	0.3422
C	87	4.41117	0.57492	0.18543
C	88	4.36672	0.5577	0.1779
C	89	4.33937	0.51484	0.25778
C	90	4.35803	0.48952	0.3374
N	1	4.0059	0.04044	0.35815
N	2	4.11439	0.26164	-0.00129
N	3	4.05081	-0.26431	0.90676
N	4	4.15775	-0.382	0.61214
N	5	4.6975	0.67851	0.45768
N	6	4.47269	0.56678	0.24942
O	1	3.8526	-0.06671	0.32352
O	2	3.84467	-0.01613	0.6048
O	3	4.07792	0.36772	0.59053
O	4	4.01701	0.31621	0.32971
O	5	4.31672	-0.28859	0.5268
O	6	4.31238	-0.34811	0.76543
O	7	4.08535	-0.12445	0.34916
O	8	4.15019	-0.11184	0.567
O	9	4.76582	0.58529	0.30204
O	10	4.811	0.64709	0.02969
O	11	4.41291	0.41395	0.49805
O	12	4.35919	0.41225	0.1487

Table S3. UHPLC gradient conditions of 2,4-D, MB, and RhB. UHPLC gradient conditions, percentage of: (A) 0.1% formic acid solution in water; (B) ACN.

Time (min)	Flow (mL/min)	A%	B%
0.0	0.3	80	20
0.2	0.3	80	20
1.5	0.3	5	95
3.0	0.3	5	95
4.0	0.3	80	20
5.0	0.3	80	20

Table S4. UHPLC gradient conditions of gentamicin. UHPLC gradient conditions, percentage of: (B) ACN; (C) 200 mM ammonium formate solution in water.

Time (min)	Flow (mL/min)	B%	C%
0.0	0.3	20	80
0.2	0.3	20	80
1.0	0.3	80	20
1.5	0.3	80	20
2.0	0.3	20	80

Table S5. Mass conditions.

Compounds	Parent ion (m/z)	Daughter ion (m/z)	Dwell	Cone (V)	Collision (eV)	Ionization mode
Gentamicin	478.4	156.7*/321.8	0.163	55	22/14	PI
2,4-D	218.7	160.9*/125.4	0.163	18	8/10	NI
MB	284.0	268.0*/240.0	0.163	30	15/20	PI
RhB	443.0	399.0*/355.0	0.163	60	50/46	PI

Table S6. The linearity of UHPLC-MS/MS method for 2,4-D, MB, RhB, and gentamicin.

Analytes	Calibration plot	Linear range (ng/mL)	R^2	LOQ [a] (ng/mL)
2,4-D	y=1771.92x+0.6786	0.05-200.0	0.9924	0.01
MB	y=322.90x–192.74	0.1-200.0	0.9974	0.1
RhB	y=41010.1x–2040.6	0.05-200.0	0.9940	0.01
Gentamicin	y=158.25x–155.84	0.1-200.0	0.9918	0.1

[a] Limit of quantitation. S/N ratio = 10.
